# Occupational Diffuse Alveolar Hemorrhage Due to Metal Fume and Nitric Acid Exposure: A Case Report

**DOI:** 10.7759/cureus.70618

**Published:** 2024-10-01

**Authors:** Mototaka Hattori, Akimichi Nagashima, Keiko Abe, Shogo Kasai, Yasumi Okochi

**Affiliations:** 1 Department of Respiratory Medicine, Japan Community Healthcare Organization Tokyo Yamate Medical Center, Tokyo, JPN; 2 Department of Pathology, Japan Community Healthcare Organization Tokyo Yamate Medical Center, Tokyo, JPN

**Keywords:** bronchoalveolar lavage, diffuse alveolar hemorrhage, metal fumes, nitric acid, occupational lung disease, systemic steroids

## Abstract

A 52-year-old male welder, who had been exposed to metal fumes and nitric acid without adequate protective measures, presented with symptoms of cough, dyspnea, and fever. He was admitted to our hospital, where bronchoscopy revealed hemorrhagic bronchoalveolar lavage fluid. He was diagnosed with diffuse alveolar hemorrhage (DAH) due to occupational inhalation of metal fumes and nitric acid. Treatment with corticosteroids led to a rapid recovery without pulmonary sequelae. The case highlights the potential for metal fumes and/or nitric acid to induce DAH, the effectiveness of corticosteroids in treating the condition, and the critical importance of occupational protective measures.

## Introduction

Diffuse alveolar hemorrhage (DAH) is a life-threatening syndrome that leads to respiratory failure. It often presents with severe symptoms, and the typical clinical manifestations include hemoptysis, anemia, diffuse radiographic pulmonary infiltrates, and hypoxemic respiratory failure. Collagen vascular disease remains the primary cause of DAH, although drugs, environmental dust, and infections can also trigger this condition [1]. DAH is an uncommon manifestation of occupational lung disease [2], which usually presents with acute reactions, such as fume-induced asthma, acute bronchitis, chemical pneumonia, or hypersensitivity pneumonitis, or with delayed manifestations, such as chronic bronchitis, pneumoconiosis, or lung cancer.

In this case, we present a rare case of occupational DAH caused by exposure to metal fumes and nitric acid. The typical clinical course and management strategies of occupational DAH have not been fully studied. This report aims to add valuable insights to improve the understanding of the characteristics of occupational DAH.

## Case presentation

A 52-year-old man presented to the hospital with a chief complaint of cough, sputum production, and dyspnea. He was a smoker with a 20 pack-year history but had no previous lung disease. Around a month before the onset of his symptoms, he had changed jobs and was assigned tasks involving chemical etching and welding. During his work, he was exposed to the inhalation of metal fumes and nitric acid without wearing an appropriate face mask. Initially, over-the-counter medications provided temporary relief, but the progression of symptoms, including cough and fever, led him to seek hospital consultation and subsequent admission for further evaluation.

On admission, his dyspnea had become severe. Physical examination revealed tachycardia, tachypnea, bilateral coarse crackles, and impaired oxygenation with a low pulse oxygen saturation of 88% on room air. He did not produce bloody sputum. Initial blood tests indicated a white blood cell (WBC) count of 9,820/µL, hemoglobin of 14.2 g/dL, and C-reactive protein (CRP) of 0.9 mg/dL. No significant autoantibodies were detected. Severe acute respiratory syndrome coronavirus 2 (SARS-CoV-2) polymerase chain reaction analyses returned a negative result (Table 1).

**Table 1:** Laboratory values AST, aspartate aminotransferase; ALT, alanine aminotransferase; CRP, c-reactive protein; ANA, antinuclear antibody; PR3-ANCA, proteinase 3 anti-neutrophil cytoplasmic antibody; MPO-ANCA, myeloperoxidase anti-neutrophil cytoplasmic antibody; GBM, glomerular basement membrane; PT, prothrombin time; PT-INR, prothrombin time-international normalized ratio; APTT, activated partial thromboplastin time; SARS-CoV-2, severe acute respiratory syndrome coronavirus 2

Parameter	Value	Reference range
White blood cell count	9.8 × 10^3^/µL	3.5–9.0 × 10^3^/µL
Neutrophils	80.7%	37.0–72.0%
Eosinophils	2.4%	0.0–5.0%
Hemoglobin	14.2 g/dL	14.0–18.0 g/dL
Platelet count	45.8 × 10^4^/µL	12.0–36.0 × 10^4^/µL
AST	16 U/L	10-33 U/L
ALT	31 U/L	4-30 U/L
Creatinine	0.69 mg/dL	0.65–1.07 mg/dL
Blood urea nitrogen	11 mg/dL	8–20 mg/dL
Sodium	140 mEq/L	135–145 mEq/L
Potassium	3.8 mEq/L	3.4–5.0 mEq/L
Albumin	3.8 g/dL	3.9–4.9 g/dL
CRP	0.9 mg/dL	0.0–0.4 mg/dL
Beta-D glucan	≤4 pg/mL	<11.0 pg/mL
ANA	<1:40	<1:40
PR3-ANCA	<1.0 U/mL	<3.5 U/mL
MPO-ANCA	<1.0 U/mL	<3.5 U/mL
Anti-GBM antibody	<2.0 U/mL	<3.0 U/mL
PT	12.0 sec	10.5–13.5 sec
PT-INR	1.05 INR	0.85–1.15 INR
APTT	30.5 sec	24–38 sec
SARS-CoV-2 polymerase chain reaction	Negative	Negative

**Figure 1:**
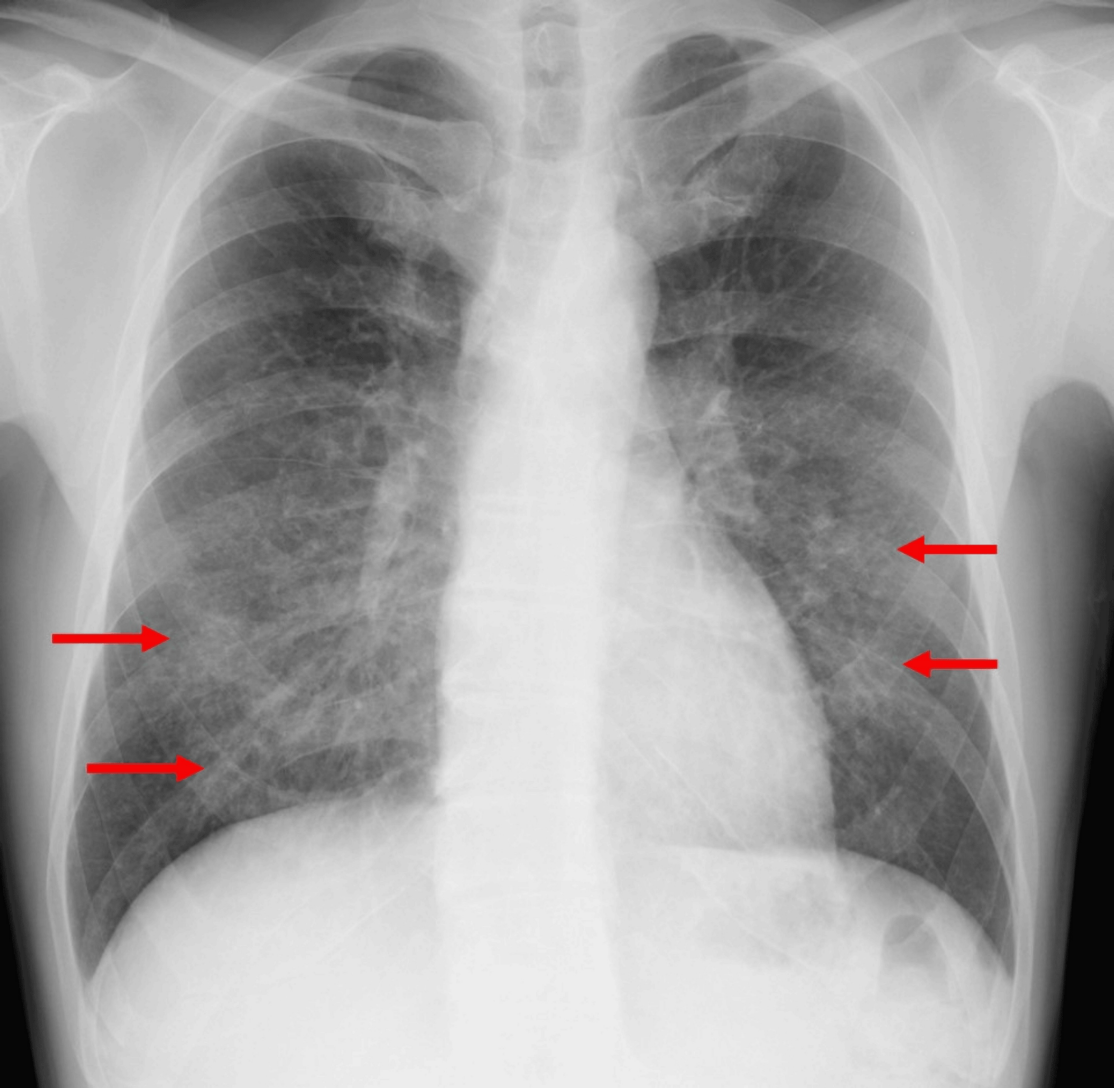
Chest X-ray showing consolidations in bilateral middle and lower lung fields (arrows).

**Figure 2:**
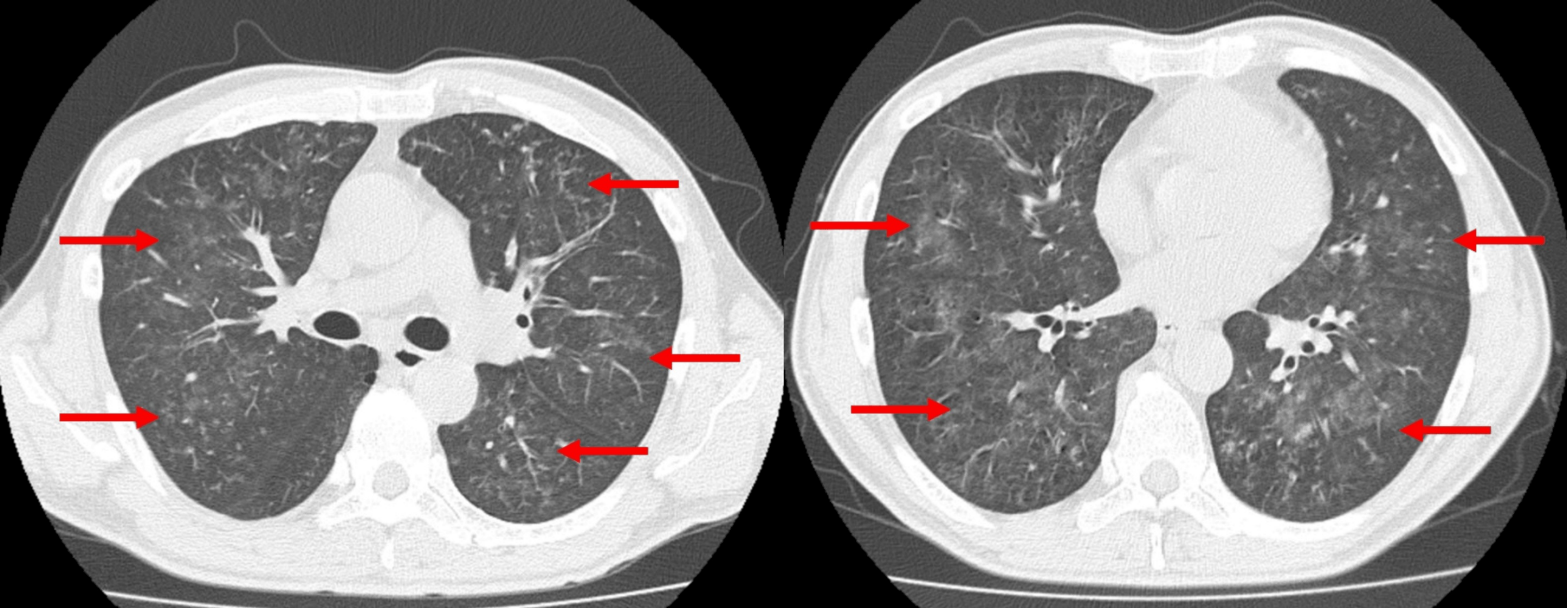
Chest computed tomography (CT) showing bilateral diffuse ground glass opacities (arrows).

**Figure 3:**
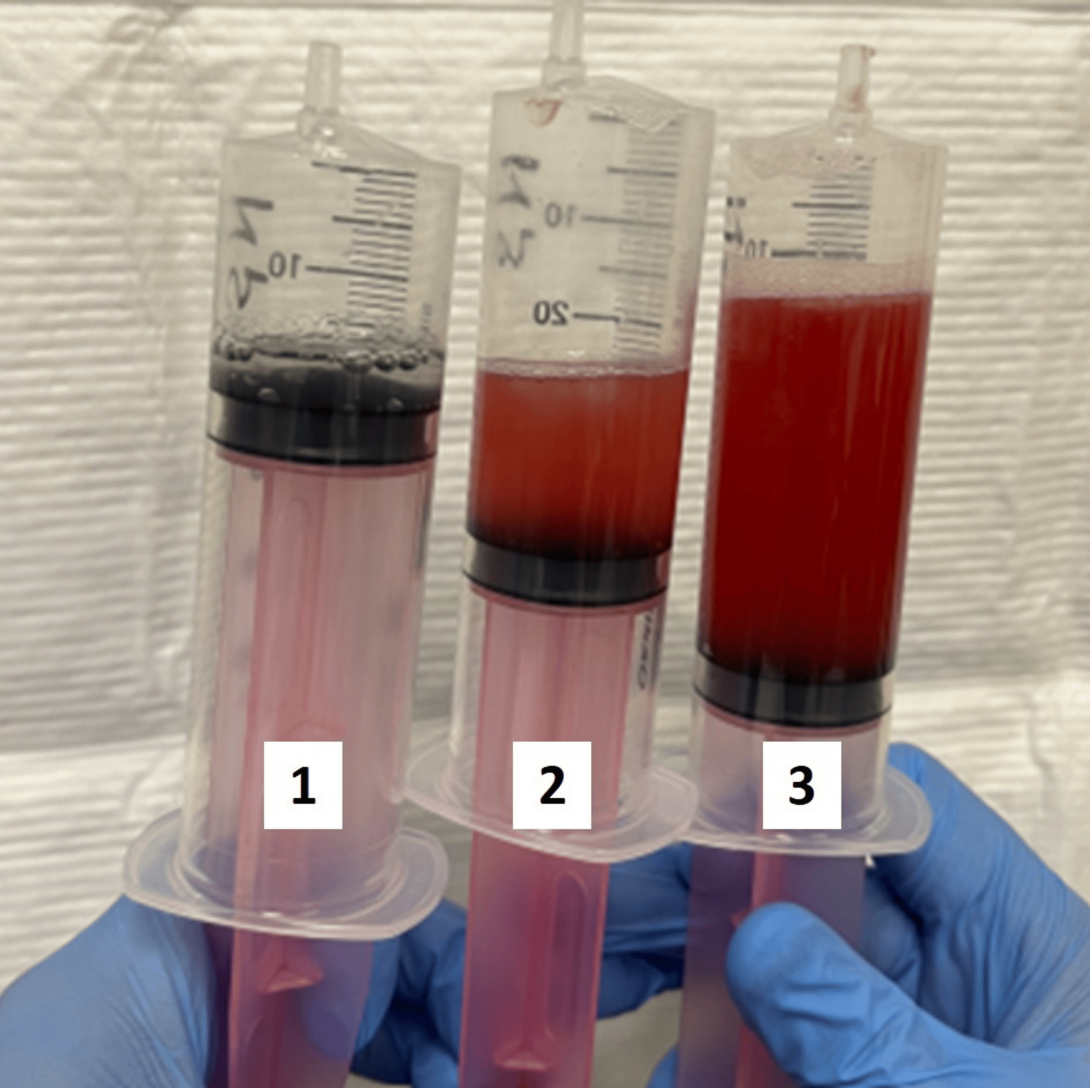
Bronchoalveolar lavage fluid showing sequential progressive hemorrhagic fluid.

Chest X-ray showed bilateral consolidation (Figure 1), and chest computed tomography (CT) scan showed centrilobular ground-glass opacities and multiple micronodules in bilateral lung fields (Figure 2). Despite the initiation of ceftriaxone treatment, the patient’s symptoms and respiratory status progressively worsened. A bronchoscopy was performed, and bronchoalveolar lavage fluid (BALF) was obtained from the lateral bronchus, revealing progressively hemorrhagic fluid characteristic of alveolar hemorrhage (Figure 3). BALF analysis showed a high cellular fraction with 48% lymphocytes, and cytology revealed hemosiderin-laden macrophages on iron staining. Microbiological analysis of samples revealed no significant bacterial evidence, and no fungal or mycobacterial isolates were identified.

It was noted that the patient had not used a face mask and had directly inhaled fumes during his work. Based on these findings, we diagnosed him with DAH following inhalation exposure to metal fumes and nitric acid. Systemic corticosteroid therapy was initiated with 1 g of methylprednisolone for three consecutive days following bronchoscopy, followed by 30 mg of prednisolone, which led to an immediate recovery from respiratory failure. Subsequently, the dosage of prednisolone was gradually tapered. To date, the patient’s condition has remained stable without recurrence of DAH.

## Discussion

DAH is a clinical syndrome characterized by blood-filled alveolar spaces due to compromised alveolar capillaries, which can sometimes lead to fatal consequences. The diagnosis of DAH relies on a thorough assessment of the medical history, blood test results, and radiographic findings. A distinction should be made between the immunogenic and nonimmunogenic causes of DAH. The most common immunogenic cause is reported to be vasculitis, followed by systemic lupus erythematosus and rheumatoid arthritis [3]. Although inhalation exposure to occupational dust, such as metal and plastic fumes, has been demonstrated in case reports to lead to lung injury such as DAH, instances of metal fume inhalation inducing DAH are uncommon [4,5].

Arc welding is a technique that involves the creation of an electric arc, which produces intense heat and is used to join a coated welding rod with the base metal. During this process, the metal vaporizes at high temperatures, and as the vapor cools upon exposure to air, it condenses into fine solid particles, forming metal fumes [6]. Inhalation of these fumes can lead to various respiratory diseases. Although the precise mechanisms by which metal fumes induce DAH remain unclear, a previous study demonstrated that nonsmokers who were exposed to welding fumes immediately developed a significant increase in WBC and neutrophil counts [7]. Furthermore, elevated levels of inflammatory cytokines, such as interleukin (IL)-1β and tumor necrosis factor (TNF)-α, were observed in the lung tissues from arc welders [8]. In our patient, we considered that welding fumes might have induced inflammatory damage to the bronchial epithelium and ultimately caused DAH. The patient was also involved in wet etching, which likely caused the inhalation of nitric acid and nitrogen oxide.

Nitric acid is frequently used for etching and as a cleanser in industrial manufacturing. The products of nitric acid thermal decomposition include nitric oxide and nitrogen oxide. Nitrogen oxide is a free radical that leads to cell membrane lipid peroxidation and damage of the cell membranes, cells, and intercellular tight junctions, mainly in the bronchial and type 1 alveolar epithelial cells and vascular endothelial cells, ultimately causing increased permeability [9]. There have been reports of pulmonary edema and acute respiratory distress syndrome secondary to nitric acid [9,10]. However, we did not find any reported cases of DAH secondary to the inhalation of nitric acid and nitrogen oxide. In our case, the patient had inhalation exposure to metal fumes, which were generated during the welding of magnesium plates, and to nitric acid during the wet etching process at a photoengraving factory. It was difficult to determine whether the cause of DAH was inhalation of metal fumes, nitric acid, nitrogen oxide, or a combination of these factors.

The approach to DAH treatment depends on the underlying cause. In cases of DAH associated with collagen diseases or drug-induced reactions, immunosuppressive therapy with steroids is often employed [11]. Although avoidance of metal fume inhalation alone was previously reported to alleviate hemoptysis [5], the condition of our patient deteriorated, with worsening symptoms and respiratory failure, despite such measures. Therefore, we administered steroid treatment, which resulted in a favorable response. Considering the involvement of cytokine production in the development of DAH, steroid treatment might have alleviated the inflammation in the respiratory tract and consequently improved the condition [11]. Although the prevention of inhalation exposure to harmful substances is the primary approach for occupational DAH, steroid treatment may be considered if the respiratory status worsens. Further investigation is necessary to elucidate the specific management and treatment of occupational DAH.

## Conclusions

We reported a case of DAH in an individual who recently transitioned to a welding job. This case demonstrates the potential efficacy of corticosteroids as a treatment for occupational DAH.

## References

[1] Lara AR, Schwarz MI (2010). Diffuse alveolar hemorrhage. Chest.

[2] Reid PA, Reid PT (2013). Occupational lung disease. J R Coll Physicians Edinb.

[3] Alexandre AT, Vale A, Gomes T (2019). Diffuse alveolar hemorrhage: how relevant is etiology?. Sarcoidosis Vasc Diffuse Lung Dis.

[4] Choudhari OK, Ojha UC, Gothi D, Spalgais S, Singh P, Rani A (2020). Diffuse alveolar hemorrhage secondary to plastic fume exposure: a case report. Monaldi Arch Chest Dis.

[5] Uzun O, İnce O, Bakalov V, Tuna T (2012). Massive hemoptysis due to welding fumes. Respir Med Case Rep.

[6] Zeidler-Erdely PC, Erdely A, Antonini JM (2012). Immunotoxicology of arc welding fume: worker and experimental animal studies. J Immunotoxicol.

[7] Kim JY, Chen JC, Boyce PD, Christiani DC (2005). Exposure to welding fumes is associated with acute systemic inflammatory responses. Occup Environ Med.

[8] Andujar P, Simon-Deckers A, Galateau-Sallé F (2014). Role of metal oxide nanoparticles in histopathological changes observed in the lung of welders. Part Fibre Toxicol.

[9] Murphy CM, Akbarnia H, Rose SR (2010). Fatal pulmonary edema after acute occupational exposure to nitric acid. J Emerg Med.

[10] Kao SL, Yap ES, Khoo SM, Lim TK, Mukhopadhyay A, Teo ST (2008). Acute lung injury after inhalation of nitric acid. Eur J Emerg Med.

[11] Park JA (2021). Treatment of diffuse alveolar hemorrhage: controlling inflammation and obtaining rapid and effective hemostasis. Int J Mol Sci.

